# Effect of nonionic and amphoteric surfactants on salivary pellicles reconstituted in vitro

**DOI:** 10.1038/s41598-021-92505-4

**Published:** 2021-06-21

**Authors:** Hannah Boyd, Juan F. Gonzalez-Martinez, Rebecca J. L. Welbourn, Kun Ma, Peixun Li, Philipp Gutfreund, Alexey Klechikov, Thomas Arnebrant, Robert Barker, Javier Sotres

**Affiliations:** 1 Biomedical Science Department, Biofilms-Research Center for Biointerfaces, Malmö University, 20506 Malmö, Sweden; 2 ISIS Neutron and Muon Source, Rutherford Appleton Laboratory, Didcot, OX11 0QX UK; 3 Institut Laue Langevin, 71 Avenue des Martyrs, 38000 Grenoble, France; 4 Department of Physics and Astronomy, Uppsala University, 75120 Uppsala, Sweden; 5 School of Physical Sciences, University of Kent, Canterbury, CT2 7NZ UK

**Keywords:** Physical chemistry, Surface chemistry, Dental biomaterials, Biophysical chemistry, Biological physics

## Abstract

Surfactants are important components of oral care products. Sodium dodecyl sulfate (SDS) is the most common because of its foaming properties, taste and low cost. However, the use of ionic surfactants, especially SDS, is related to several oral mucosa conditions. Thus, there is a high interest in using non-ionic and amphoteric surfactants as they are less irritant. To better understand the performance of these surfactants in oral care products, we investigated their interaction with salivary pellicles i.e., the proteinaceous films that cover surfaces exposed to saliva. Specifically, we focused on pentaethylene glycol monododecyl ether (C_12_E_5_) and cocamidopropyl betaine (CAPB) as model nonionic and amphoteric surfactants respectively, and investigated their interaction with reconstituted salivary pellicles with various surface techniques: Quartz Crystal Microbalance with Dissipation, Ellipsometry, Force Spectroscopy and Neutron Reflectometry. Both C_12_E_5_ and CAPB were gentler on pellicles than SDS, removing a lower amount. However, their interaction with pellicles differed. Our work indicates that CAPB would mainly interact with the mucin components of pellicles, leading to collapse and dehydration. In contrast, exposure to C_12_E_5_ had a minimal effect on the pellicles, mainly resulting in the replacement/solubilisation of some of the components anchoring pellicles to their substrate.

## Introduction

Surfactants are a common ingredient of oral hygiene products like toothpastes and mouthwashes^1,2^. They act as solubilizing, dispersing and wetting agents. Moreover, they promote foaming, which is preferred by consumers since it provides a perception of cleanliness^3^. Sodium dodecyl sulfate (SDS) is the most common surfactant present in oral products since the development of synthetic surfactants^2^, nowadays being one of the most commonly used surfactants in oral health products at concentration of up to 2%^4^. However, the use of ionic surfactants like SDS has been related to exacerbation of periodontal disease, initiation and progression of gingival recession and recurrent oral ulceration^5^. In this regard, non-ionic and amphoteric surfactants attract significant interest as they have been shown to be less irritant^6^. While it is difficult to compete against the use of SDS because of its foaming properties, taste and low cost, amphoteric surfactants, especially cocamidopropyl betaine (CAPB) are becoming more popular in personal care products, including toothpastes^7^. Nonionic surfactants are instead nowadays preferred in mouthwashes^1^. The goal of this work was to increase the scientific basis of the mechanisms of action of the less-irritant non-ionic and amphoteric surfactants so that the formulation of oral care product could be improved. Specifically, we have focused on the effect that these two types of surfactants have on reconstituted salivary pellicles.

Salivary pellicles are thin (thickness of few nanometers) organic (mostly proteinaceous) films that form rapidly on oral surfaces upon exposure to saliva^8^. Salivary pellicles fulfil many functions. These include e.g., regulation of tooth mineralization and demineralization processes, lubrication, hydration, acting as a diffusion barrier and buffering ability^8,9^. Thus, it is reasonable that the use of oral products with minimal effect on salivary pellicles would also be gentler towards oral mucosa surfaces. Indeed, ionic surfactants, especially SDS, are known to highly damage salivary pellicles^10,11^. This agrees with the fact that electrostatic interactions have been shown to play an important role in pellicle structure^12,13^. In this work we explored whether the less-irritant nonionic and amphoteric surfactants exhibit different interactions with salivary pellicles than ionic surfactants.

For this purpose, we investigated salivary pellicles reconstituted on model solid surfaces before and after exposure to a nonionic surfactant (pentaethylene glycol monododecyl ether, C_12_E_5_) and an amphoteric surfactant (cocamidopropyl betaine, CAPB) by means of different surface techniques i.e., quartz crystal microbalance with dissipation (QCM-D), null-ellipsometry, force spectroscopy and neutron reflectometry. All of these techniques have already proven to be highly useful for pellicle structure investigations^4,13–15^. QCM-D, NR and ellipsometry, require highly planar, macroscopic and, for the latter, reflective solid surfaces. Because of these requirements, it was not possible to use more relevant oral cavity substrates e.g., oral mucosa and enamel, to reconstitute salivary pellicles. An important factor to consider for choosing the right model surface is its charge, as this is known to play an important role on the formation of salivary pellicles^16,17^. Oral mucosa surfaces are decorated with the MUC1 mucin^18,19^. Thus, they have an anionic character. A similar character has been reported for hydroxyapatite^20^, the main component of enamel. Therefore, we employed in our experiments silica surfaces, which also have an anionic character^21^, as substrate for salivary pellicles.

Overall, we show that both C_12_E_5_ and CAPB are gentler towards salivary pellicles reconstituted on silica surfaces than SDS, which removes them completely. However, CAPB and C_12_E_5_ affected the reconstituted pellicles to different extents and by means of different mechanisms.

## Results

### Quartz crystal microbalance with dissipation (QCM-D) characterization

**Figure 1:**
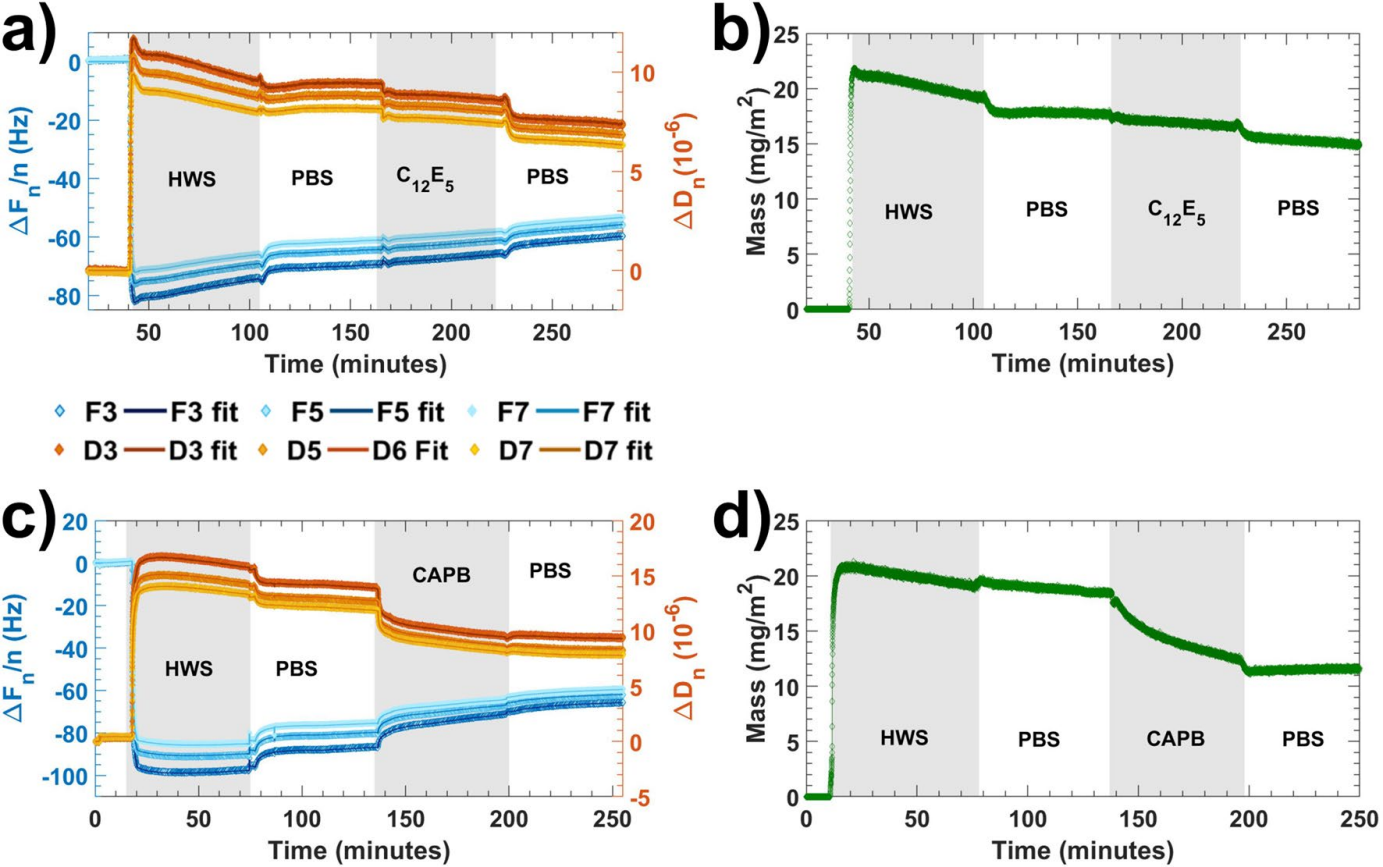
**(a)** QCM-D data (markers) and corresponding fits to the Voigt model using overtones 3rd, 5th, an 7th (solid lines) for a representative experiment where human whole saliva (HWS) was adsorbed on silica surfaces, rinsed with PBS buffer, exposed to C_12_E_5_ in PBS solution and finally rinsed with PBS buffer again. (**b)** Adsorbed mass calculated from fitting the data in **(a)** to the Voigt model. (**c)** QCM-D data (markers) and corresponding fits to the Voigt model using overtones 3rd, 5th, an 7th (solid lines) for a representative experiment where human whole saliva (HWS) was adsorbed on silica surfaces, rinsed with PBS buffer, exposed to a CAPB in PBS solution and finally rinsed with PBS buffer again. (**d)** Adsorbed mass calculated from fitting the data in (c) to the Voigt model.

Raw QCM-D data, corresponding fits to the Voigt viscoelastic model and areal masses obtained from these fits, from representative experiments showing the formation of salivary pellicles on hydrophilic silica surfaces, and their subsequent exposure to C_12_E_5_ and CAPB in PBS solutions are shown in Fig. 1.

QCM-D data confirmed the formation of viscoelastic (as inferred from the high dissipation shift values) salivary pellicles upon the exposure of the silica substrates to saliva in a time frame of minutes in agreement with previous reports^13,15^. Subsequent exposure to both C_12_E_5_ and CAPB led to a decrease in both frequency and dissipation shifts. However, these quantities did not return to zero values indicating that the investigated surfactants did not entirely remove the pellicles. This is in contrast with the effect of SDS, which completely removes pellicles formed on hydrophilic surfaces (^11^, Supplementary Information Section S4).

The specific effect on QCM-D signals was different for the two investigated surfactants. Data registered at the end of the PBS buffer rinsing steps performed before and after exposure to surfactant solutions indicated a decrease in frequency shift of ~ 7 Hz in the case of C_12_E_5_ (Fig. 1a) and of ~ 18 Hz in the case of CAPB (Fig. 1c), with changes in dissipation shifts following the same trend. Areal masses calculated from Voigt model fits revealed the same tendency i.e., exposure to C_12_E_5_ resulted in a decrease of ~ 3 mg·m^−2^ (Fig. 1b) while exposure to CAPB resulted in a decrease of ~ 7 mg·m^−2^ (Fig. 1d). The ratio, averaged over 3 different experiments, between QCM-D mass measured after and before exposure to surfactants evidenced the same behaviour (Fig. 3).

### Ellipsometry characterization and comparison with QCM-D results

**Figure 2:**
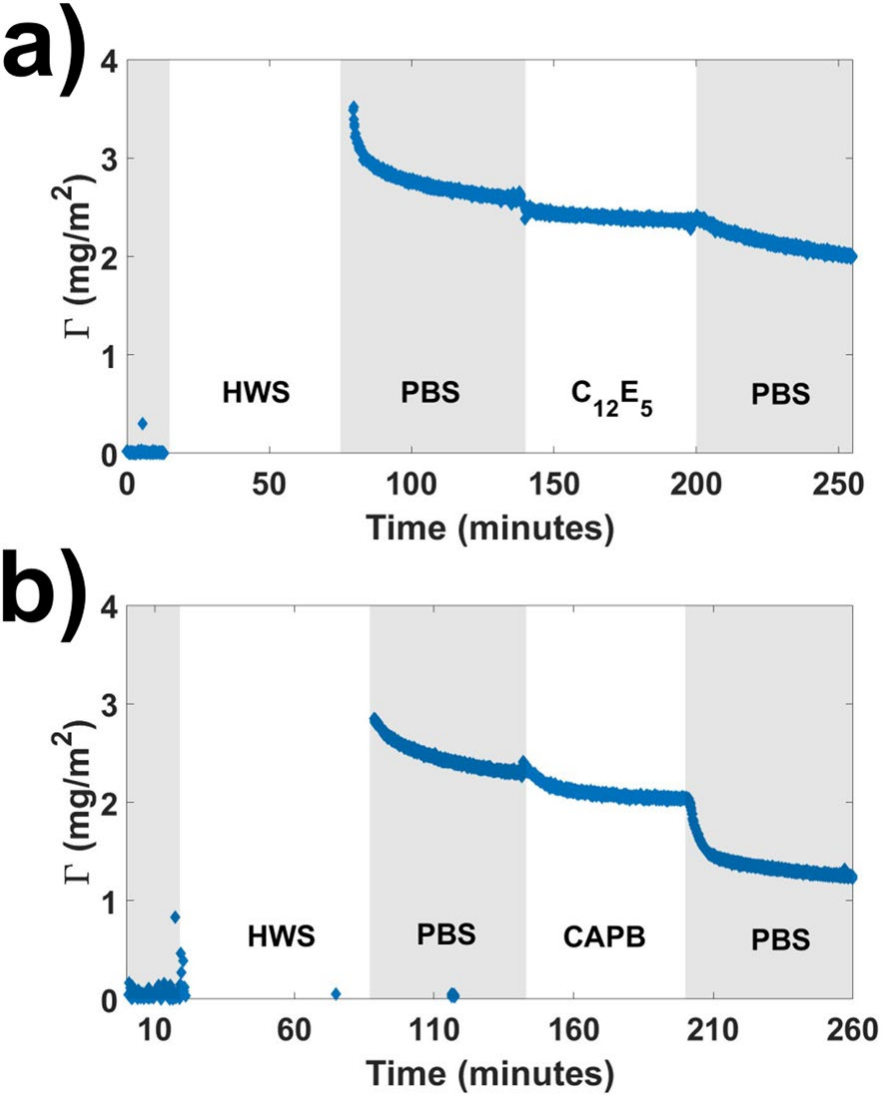
Areal mass calculated from representative ellipsometry experiments where salivary pellicles were formed, rinsed with PBS and subsequently exposed to (**a)** C_12_E_5_ and (**b)** CAPB solutions followed by a final PBS rinsing step.

**Figure 3:**
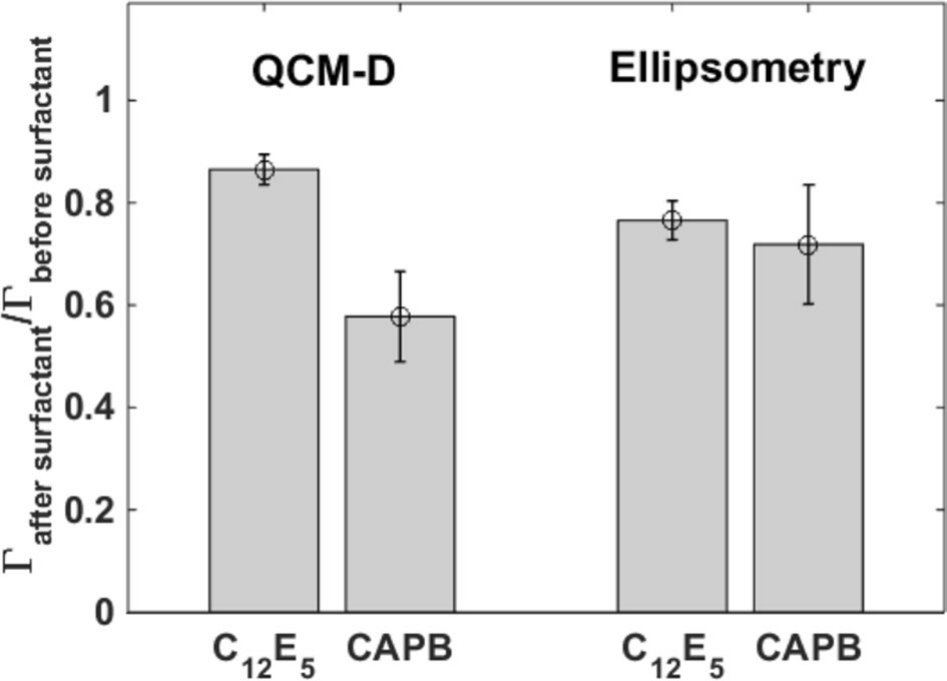
Comparison between the adsorbed mass as measured by QCM-D and ellipsometry. The mean ratio of the mass of the pellicle before to the mass after treatment by the surfactants over three data sets are shown. Error bars correspond to standard deviation values.

Areal masses from representative ellipsometry experiments where salivary pellicles were exposed to C_12_E_5_ and CAPB solutions are shown in Fig. 2. Corresponding refractive index and thickness values for these experiments are shown in Supplementary Information S5. Up to some extent, CAPB caused a larger decrease in both the areal mass and thickness of salivary pellicles compared to C_12_E_5_. However, as shown in Fig. 3, when averaged over data from different experiments the difference in amount desorbed by each surfactant was not as significant as that shown by QCM-D.

Figure 3 shows the ratio of both ellipsometry and QCM-D areal masses of salivary pellicles measured in PBS after and before surfactant exposure averaged over three different data sets. According to QCM-D, less than ~ 60% of the pellicle mass is remaining after treatment with CAPB compared to ~ 87% after C_12_E_5_ exposure. However, according to ellipsometry the difference is much lower with only ~ 5% more mass being removed by CAPB compared to C_12_E_5_. Although differences between experimental techniques are to be expected due to different sensitivities and environments, for the specific comparison of these two techniques differences are expected (as discussed later on) as ellipsometry is basically sensitive to the solid content of adsorbed layers (often called “dry mass”) whereas QCM-D is also sensitive to the mass of solvent coupled to the layers (thus, the mass monitored by QCM-D is often referred as “wet mass”).

### Force spectroscopy characterization

**Figure 4:**
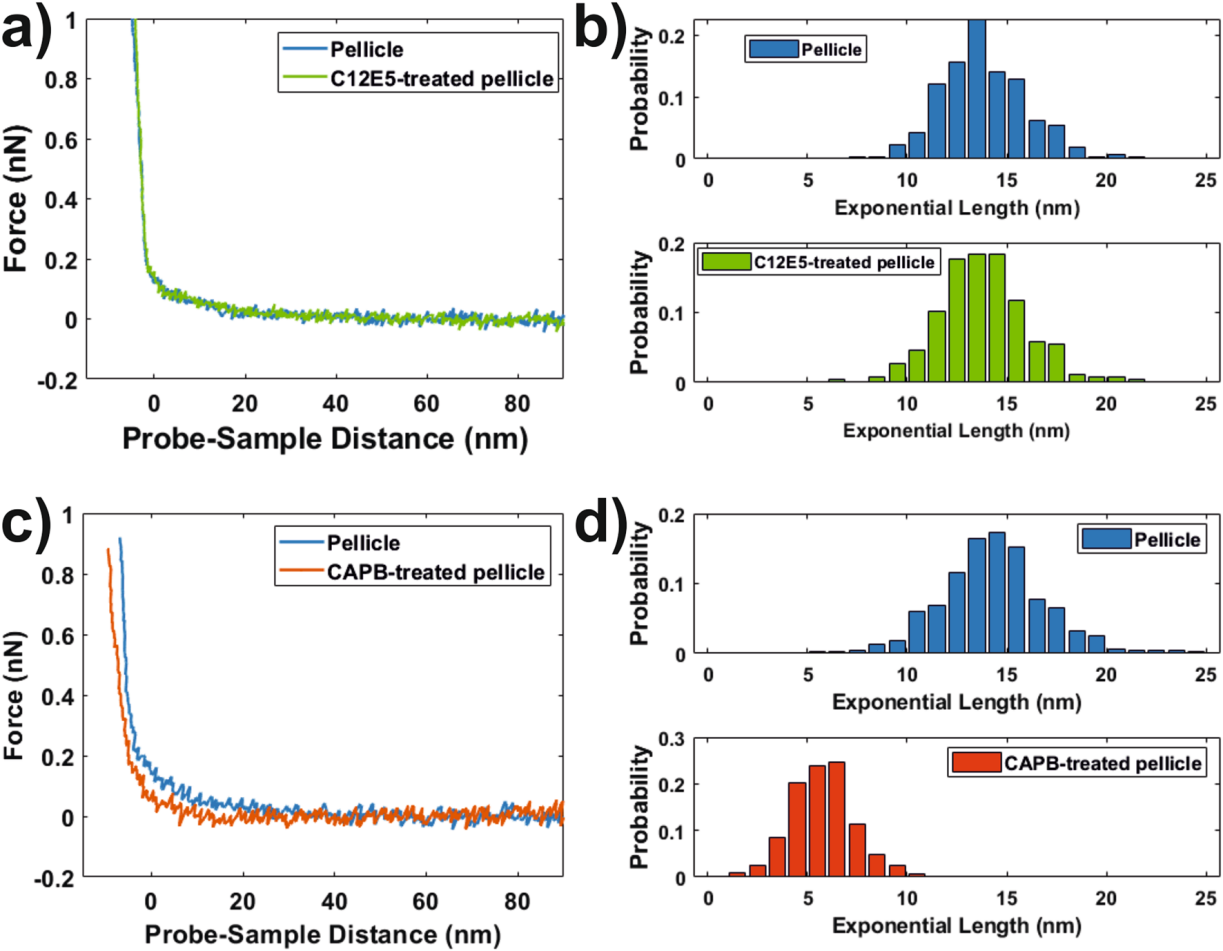
**(a)** Force vs probe-sample distance ramps on salivary pellicles (reconstituted on silica substrates) before (blue) and after (green) exposure to C_12_E_5_ for 1 h and subsequently rinsed with PBS buffer. (**b)** Probability distributions for the characteristic length obtained from exponential fits of the non-contact regions of force ramps on pellicles before and after exposure to C_12_E_5_. (**c)** Force vs probe sample distance ramps on salivary pellicles (reconstituted on silica substrates) before (blue) and after (red) exposure to CAPB for 1 h and subsequently rinsed with PBS buffer. (**d)** Probability distributions for the characteristic length obtained from exponential fits of the non-contact regions of force ramps on pellicles before and after exposure to CAPB.

Salivary pellicles before and after exposure to C_12_E_5_ and CAPB solutions were investigated by means of AFM-based force spectroscopy. Data from a representative experiment are shown in Fig. 4. Force ramps (representative examples shown in Fig. 4a,c) from all experiments (pellicles before and after exposure to both surfactants) exhibited long-range exponential-like repulsive forces, characteristic for steric interactions, when mechanically confining the samples with the AFM probe. When confined enough, the force-distance behaviour drastically changed to one that could be modelled by the Hertz mechanical contact model instead (specifics on the fitting procedure are detailed in Supplementary Information Section S3). Interestingly, exposure to C_12_E_5_ did not have a significant effect on the repulsive long-range steric forces (Fig. 4a). This was clearly seen in the characteristic length, λ_exp_ (Eq. 2), found when fitting to exponential functions the region of the ramps where these forces were observed (Fig. 4b).

For CAPB-treated pellicles, imaging of the samples by means of AFM revealed (Supplementary Information Section S6) that a fraction of ~ 5% of the overall surface area became covered with a µm-domains (absent before exposure to this surfactant) of a material that we hypothesized to be aggregated CAPB. For force spectroscopy experiments on these samples, we avoided areas covered by these aggregates. Force ramps performed on the remaining homogeneous CAPB-treated pellicle (accounting for ~ 95% of the surface area) revealed that exposure to this amphoteric surfactant drastically lowered, by a factor ~ 3, the distances to which the repulsive steric forces opposing mechanical confinement extended (Fig. 4c,d).

### Neutron reflectivity characterization

To further investigate the mechanisms by which C_12_E_5_ and CAPB interact with reconstituted salivary pellicles, neutron reflectivity (NR) was used. The non-destructive and highly penetrating nature of neutron beams allows extracting structural properties perpendicular to the surface of films, providing high resolution information on layer thickness, interface roughness, and layer density (in terms of hydration and scattering length density, a quantity equivalent to a refractive index that is determined by the composition of specific regions of the sample).

Each of the silicon blocks used as pellicle substrates were first characterized in three different contrasts: PBS buffer prepared with H_2_O (hPBS, SLD = − 0.56 × 10^–6^ Å^−2^), with silicon matched water (0.38:0.62 D_2_O:H_2_O, smwPBS, SLD = 2.07 × 10^–6^ Å^−2^) and with D_2_O (dPBS, SLD = 6.36 × 10^–6^ Å^−2^). Similar characterizations were performed after formation of salivary pellicles on each block and after treatment of the pellicles with the surfactants (except for CAPB-treated surfactants, for which only dPBS and hPBS characterizations were performed). Specifically, we report here experiments on salivary pellicles treated with chain deuterated C_12_E_5_ (dC_12_E_5_), with fully hydrogenated C_12_E_5_ (hC_12_E_5_) and with hydrogenated CAPB.

For NR data analysis, the refnx software was used^22^. In all experiments, the interface between the silicon support and the bulk solvent was considered as a stratified medium composed by different slabs. The reflectivity originating from such an interface can be described according to the optical matrix method^23^, where each of the slabs included in the model is characterized by four output parameters: the layer thickness, the scattering length density (SLD), the volume fraction of solvent (hydration) and the surface roughness. In all cases, fits of NR for each of the investigated systems were co-refined using data from all contrasts available. However, while similar, different salivary pellicles exhibited slight differences that did not allow to co-refine fits from different pellicles i.e., it was not possible to simultaneously fit reflectivities from different pellicles with identical fitting parameters (even though similar results as those reported in ^12^ were obtained in all cases), and, therefore, from pellicles treated with dC_12_E_5_ and hC_12_E_5_.

**Figure 5:**
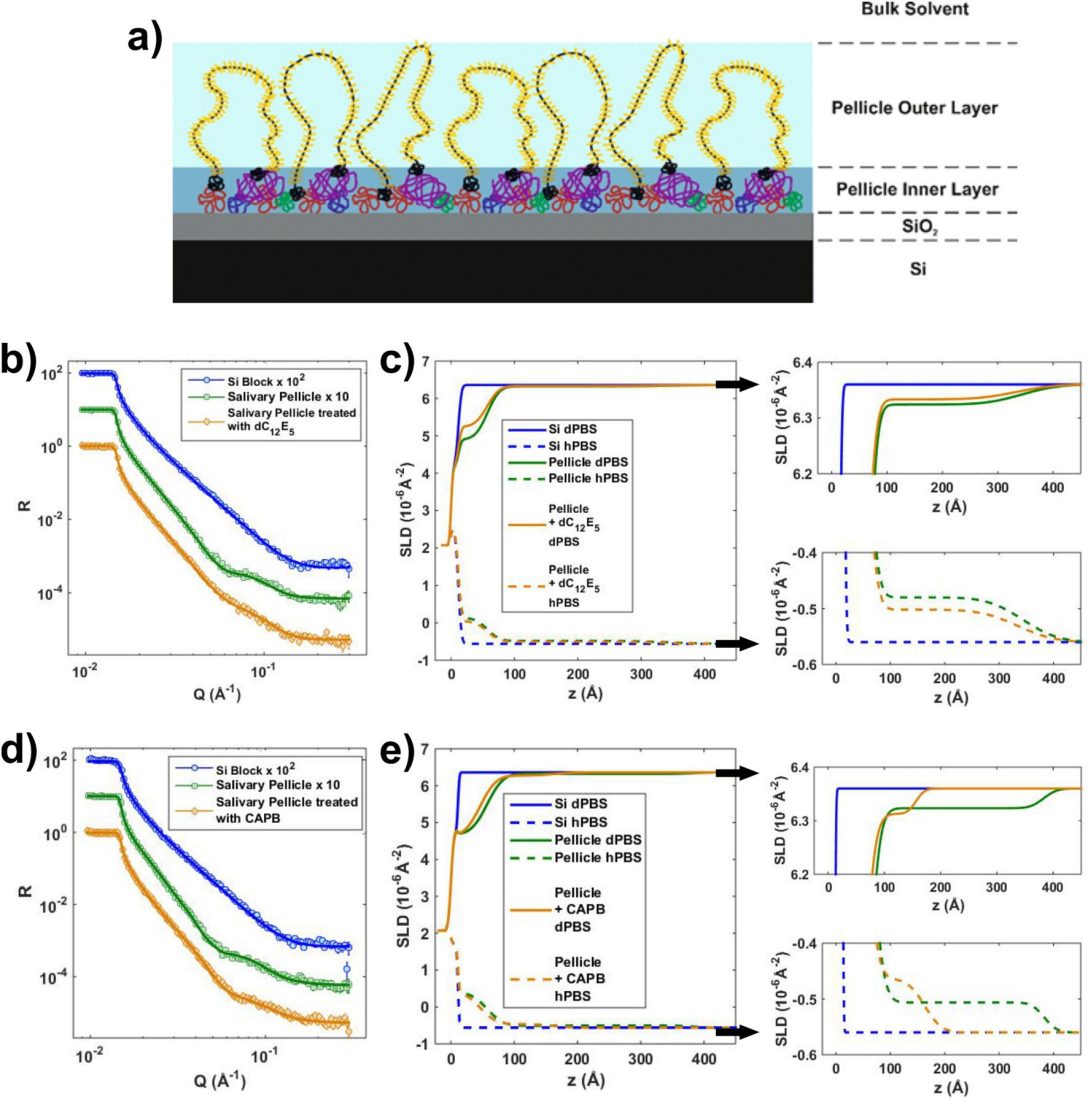
**(a)** Illustration of the two-layered pellicle model used to fit NR data (illustration created with CorelDRAW, Corel Corporation, Canada). (**b)** NR curves, in dPBS contrast, and corresponding fits (solid lines) for a clean silicon block and a pellicle adsorbed on that block before and after treatment with dC_12_E_5_. (**c)** Corresponding SLD profile for both dPBS and hPBS contrasts. On the right hand side, zooms for the regions corresponding to the pellicle outer layer are shown. (**d)** NR curves, in dPBS contrast, and corresponding fits (solid lines) for a clean silicon block and a pellicle adsorbed on that block before and after treatment with CAPB. (**e)** Corresponding SLD profiles for both dPBS and hPBS contrasts. On the right hand side zooms for the regions corresponding to the pellicle outer layer are shown.

For fitting NR data on the clean silicon blocks, a simple Si/SiO_2_/Solvent model was used. For fitting NR data obtained for salivary pellicles, we used a two-layered structure for the pellicles (Fig. 5a) in agreement both with previous NR studies^12,16^ and with a model reported in multiple other studies where pellicles consist of an inner thin dense layer and an outer thick diffuse layer^13,16,24,25^. For fitting NR data from the pellicles after being exposed to the surfactants, we used the same model allowing for changes in the inner and outer pellicle layers. Moreover, we allowed the pellicle layers to include a percentage of surfactant (Supplementary Information Section S7).

Raw and fitted NR data for a silicon block and a salivary pellicle subsequently formed on that block before and after treatment with dC_12_E_5_ are shown in Fig. 5b. Corresponding SLD profiles are shown in Fig. 5c. Similarly, raw and fitted NR data for a different silicon block and a pellicle subsequently formed on that block before and after treatment with CAPB are shown in Fig. 5d, with corresponding SLD profiles in Fig. 5e. Relevant parameters obtained from the fits are shown in Tables 1 and 2 for the effect on the pellicles of dC_12_E_5_ and CAPB respectively (the complete set of fit parameters is available in Supplementary Information Section S7).

**Table 1:** Parameters obtained from fits of NR data of a salivary pellicle adsorbed on a silicon block before and after treatment with dC_12_E_5_.

		Salivary pellicle before treatment with dC_12_E_5_	Salivary pellicle after treatment with dC_12_E_5_
Pellicle inner layer	Thickness (Å)	46 ± 1	45 ± 1
	Hydration (%)	62.0 ± 0.8	70.1 ± 0.5
	Salivary content (%)	100	92 ± 2
	Surfactant content (%)	–	8 ± 2
Pellicle outer layer	Thickness (Å)	286 ± 12	277 ± 16
	Hydration (%)	97.1 ± 0.2	98.0 ± 0.1
	Salivary content (%)	100	96 ± 5
	Surfactant content (%)	–	4 ± 5

The parameters were obtained from co-refined fits of 3 measurements of the Si block (in dPBS, smwPBS and hPBS), 3 measurements of the pellicle before exposure to C_12_E_5_ (in dPBS, smwPBS and hPBS) and 3 measurements after exposure to C_12_E_5_ (in dPBS, smwPBS and hPBS). Errors correspond to those calculated by means of the Bayesian Markov chain Monte Carlo (MCMC) approach implemented in the refnx software^22^.

**Table 2:** Parameters obtained from fits of NR data of a salivary pellicle adsorbed on a silicon block before and after treatment with CAPB.

		Salivary pellicle before treatment with CAPB	Salivary pellicle after treatment with CAPB
Pellicle inner layer	Thickness (Å)	46 ± 1	35 ± 1
	Hydration (%)	55.7 ± 0.3	54.4 ± 0.9
	Salivary content (%)	100	99.4 ± 0.7
	Surfactant content (%)	–	0.6 ± 0.7
Pellicle outer layer	Thickness (Å)	325 ± 11	117 ± 8
	Hydration (%)	97.5 ± 0.2	95.5 ± 0.5
	Salivary content (%)	100	92.0 ± 2.0
	Surfactant content (%)	–	8.0 ± 2.0

The parameters were obtained from co-refined fits of 3 measurements of the Si block (in dPBS, smwPBS and hPBS), 3 measurements of the pellicle before exposure to C_12_E_5_ (in dPBS, smwPBS and hPBS) and 2 measurements after exposure to C_12_E_5_ (in dPBS, and hPBS). Errors correspond to those calculated by means of the Bayesian Markov chain Monte Carlo (MCMC) approach implemented in the refnx software^22^.

In agreement with observations from other techniques, dC_12_E_5_ had a minor effect on the salivary pellicle. Mainly, the pellicle outer layer exhibited slight coiling and the hydration of the inner layer increased by ~ 10%. Fitting NR data from the pellicle treated with hC_12_E_5_ (Supplementary Information Section S7) revealed a similar tendency, even though in this case the hydration of the inner layer only increased by ~ 5%. The obtained SLD values might support that the pellicles retained some C_12_E_5_ both in the pellicle inner and outer layers. However, when considering the errors this data was not conclusive.

In contrast, exposing the pellicles to CAPB induced a significant coiling of the pellicle outer layer (relative decrease in thickness by a factor ~ 3, in good agreement with Force Spectroscopy data). The pellicle inner layer was hardly influenced by CAPB though. The SLD profile also suggested that, after rinsing, a higher amount of surfactant was retained, mostly by the pellicle outer layer.

## Discussion

In this work, we investigated the effect of a model nonionic surfactant, C_12_E_5_, and a model amphoteric surfactant, CAPB, with salivary pellicles reconstituted at solid–liquid interfaces. Both types of surfactants are considered to be less aggressive towards oral surfaces than the most commonly used ionic surfactants, especially SDS, in oral care products. Salivary pellicles i.e., the mostly proteinaceous films that adsorb on almost any surface upon exposure to saliva, constitute a protective barrier for oral surfaces. Thus, our aim was to investigate whether the gentler effect of nonionic and amphoteric surfactants correlated with a different effect on salivary pellicles from that of ionic surfactants. For this, we employed different surface techniques: force spectroscopy, QCM-D, null-ellipsometry and neutron reflectometry. The latter require the use of highly planar reflective surfaces, preventing the use of oral surfaces like mucosa or enamel to reconstitute salivary pellicles. For identifying a substrate that we could use instead, we considered two properties known to influence the properties of salivary pellicles: the substrate wettability^26^ and ionic character^8,25^. In vivo wettability measurements indicated a hydrophilic nature for both teeth and oral mucosa^27^ surfaces. Hydroxyapatite, the main component of enamel/teeth surface has an anionic character^20^. Because of being decorated with the MUC1 mucin^19^, the same applies to oral mucosa surfaces. Therefore, we used silica surfaces as substrates for salivary pellicles because of their hydrophilic anionic character and their suitability for the employed experimental techniques.

In agreement with previous reports^11^, we confirmed (Supplementary Information Section S4) that exposure of salivary pellicles reconstituted on silica surfaces to SDS concentrations above the CMC value led to a complete removal of salivary pellicles reconstituted on these surfaces. In contrast, the investigated nonionic and amphoteric surfactants had a gentler effect on the pellicles. However, these surfactants had different effects on the pellicles.

The areal mass of salivary pellicles before and after being exposed to both types of surfactants was investigated by ellipsometry and QCM-D. While ellipsometry is sensitive mostly to the dry content of the adsorbed material, QCM-D also is highly sensitive to the mass of the solvent coupled to the adsorbed films^28,29^. In our experiments, the ellipsometry mass of salivary pellicles before and after being exposed to C_12_E_5_ and CAPB solutions did not show significant differences (Fig. 3). However, QCM-D (Fig. 3) revealed that CAPB removed a significantly higher fraction than C_12_E_5_. This indicated that CAPB had a stronger interaction, with a highly hydrated fraction of the salivary pellicle at least. We can interpret this observation in terms of the two-layer model proposed for reconstituted salivary pellicles^12,13,16,24,25^. In this model, reconstituted pellicles consist of an inner thin dense layer (formed mainly of low molecular weight proteins) and an outer thick diffuse highly hydrated layer (mainly composed by the highly charged oral mucin MUC5B). In this scheme, it is reasonable to hypothesize that the amphoteric surfactant CAPB developed a strong interaction with the highly hydrated and charged outer pellicle layer, resulting in its coiling and the subsequent release of trapped solvent. This was supported by force spectroscopy and NR experiments. Force spectroscopy probes the pellicle outer surface. The long-range repulsive forces observed with this technique, when the salivary pellicles are mechanically confined, agree with the presence of steric forces that would counteract the increase of osmotic pressure resulting from the confinement of a diffuse polymer-like layer^30,31^. After exposure to CAPB solutions, the range (quantified in terms of the characteristic length obtained from the exponential fit of the non-contact region) to which steric forces extended decreased (Fig. 4c,d), by a factor ~ 3, suggesting a partial coiling/collapse of the pellicle. NR fits (Fig. 5d,e, Table 2) supported this observation by indicating that the pellicle outer layer collapsed by a similar factor. Moreover, because of the high hydration of the pellicle outer layer, its coiling also supported the significant decrease of the pellicle “wet” mass indicated by QCM-D experiments. The main component of the pellicle outer layer, the MUC5B mucin, comprises of a heavily O-glycosylated polypeptide chain resulting in a net negative charge at physiological pH as most of these glycans contain terminal sialic acids or sulfated sugar residues^32^. Thus, it is expected that electrostatic forces would play an important role in the interaction between the pellicle outer layer and external compounds. CAPB consists of a long hydrocarbon chain and a polar head group that contains a quaternary ammonium cation and a carboxylate. Therefore, it is reasonable to hypothesize that this surfactant would develop electrostatic interactions with the mucins in the pellicle outer layer, eventually resulting on its collapse and the subsequent release of coupled solvent. Indeed, SLD values from NR fits supported the presence of CAPB in the pellicle outer layer after being rinsed with surfactant-free buffer. However, one should be cautious with this interpretation, as it might also be the SLD change can be attributed to some specific salivary components being removed by the surfactant.

The gentler effect of C_12_E_5_ on salivary pellicles revealed by QCM-D and ellipsometry was also supported by force spectroscopy and NR data. Force spectroscopy showed that steric forces did not significantly change after exposure of pellicles to C_12_E_5_ solutions (Fig. 4a,b). This indicates a poor interaction between the nonionic C_12_E_5_ and the pellicle outer layer that was confirmed by NR data (Fig. 5b,c, Table 1). Effectively, NR indicated that that C_12_E_5_ had a very minor effect on the thickness or hydration of the pellicle outer layer. While the mucins in this layer also contain hydrophobic domains that could constitute potential interaction sites with C_12_E_5_, these sites probably form complexes with additional salivary components that prevent inter/intra-chain mucin interactions^12^, and therefore also prevent mucin-C_12_E_5_ interactions. This is in agreement with previous results suggesting a very limited interaction between C_12_E_5_ and mucins^33^. NR also suggested an increase in the hydration of the pellicle inner layer after exposure to C_12_E_5_. Regardless of whether this originated through replacement or solubilization mechanisms, which cannot be concluded from our data, NR data supports that the C_12_E_5_-induced partial pellicle removal observed in ellipsometry and QCM-D experiments would take place mainly in the inner layer. However, the fact that C_12_E_5_ had a very limited effect on the pellicle outer layer also indicated that this surfactant did not primarily affect the components of the pellicle inner layer that contribute to anchor the outer layer. Further studies would be required to identify the specific pellicle components that interact with C_12_E_5_ as well as the corresponding interaction mechanisms.

Overall, the presented experimental data show that the investigated nonionic and amphoteric surfactants interact with reconstituted salivary pellicles in a gentler way than SDS. However, the extent to which they modified the pellicles as well as the underlying mechanisms differed. The amphoteric CAPB surfactant would mainly interact, probably by means of attractive electrostatic forces, with the mucin components of pellicles, leading to their coiling/collapse and a subsequent release of trapped water. In this regard, it is worth to note that the lubricating properties of salivary pellicles and, therefore, their ability to protect from mechanical damage is often related to their highly hydrated nature^24,34^. Thus, the use of CAPB in oral care product might be in detriment of this property. In contrast, C_12_E_5_ removed a lower amount of salivary pellicles than CAPB. Moreover, exposure to C_12_E_5_ did not affect significantly the pellicle outer layer. Instead, our data indicated that the effect of this nonionic surfactant on the adsorbed pellicle mass might instead be related to the replacement or solubilization of a small fraction of the pellicle inner layer.

At this point, it is worth to discuss the limitations of this work and, subsequently, future directions to address them. As previously discussed, we chose silica as a substrate for this study as (i) it is a surface that can be used in all employed techniques that (ii) shares with both enamel and oral mucosal surfaces two physicochemical aspects known to play a key role in the adsorption of salivary components: wettability and ionic character. Thus, it is reasonable that the main observations of our work are of relevance for pellicles on oral surfaces i.e., enamel and mucosa. In this regard, it is of relevance that pellicles reconstituted in situ on enamel in the oral cavity show a similar structure than those reconstituted in vitro on silica^15^. A bigger difference might be expected between silica and mucosal surfaces. While mucosal surfaces are populated by highly hydrophilic anionic transmembrane mucins, properties shared by silica, it is also know that mucosal and enamel pellicles exhibit some differences with respect to structure and composition^35^. For instance, it has been shown that the presence of the transmembrane mucin MUC1 expressed by oral epithelium cells enhances the binding of salivary components, specifically MUC5B mucins^18^. Studies on e.g., MUC1 coated silica substrates could be approached by means of the techniques used in this work and would provide further insights into the interaction of pellicles with different types of surfactants. Another aspect to consider of this work is that we investigated pellicles reconstituted in vitro. The study of pellicles formed in situ might have given additional information, since they differ from those formed in vitro^36,37^. In this regard, pellicles go through a maturation process when exposed to bulk saliva in the oral cavity for long periods. However, many studies have also shown that pellicles formed after short times e.g., minutes, already fulfill protective functions up to a high degree^38^. We focused our study on pellicles reconstituted in vitro as reconstitution in situ was not an option for most of the techniques employed. Nevertheless, in order to minimize the limitations of in vitro studies, we always employed fresh saliva to reconstitute the pellicles. Pellicle-surfactant interactions would undoubtedly benefit from investigations by means of techniques that allow working with pellicles formed in situ. Among them, Transmission Electron Microscopy (TEM) has been proved as a powerful technique for structural investigations of pellicles. For instance, TEM has been successfully used to determine how the structure of pellicles reconstituted in situ are modified by a variety of mechanical and chemical challenges^39^. Finally, a natural follow-up of the present study would be to investigate bacterial attachment on pellicles previously exposed to nonionic and amphoteric surfactants.

## Methods

### Chemicals

Cocamidopropyl betaine (CAPB) was purchased from Elemental SRL (Ref. M-1247-0.5). Pentaethylene glycol monododecyl ether (C_12_E_5_) and sodium dodecyl sulfate (SDS) were purchased from Sigma-Aldrich (Refs. 76437 and 436143). Chain deuterated C_12_E_5_ was provided by the ISIS deuteration facility. All water used was of ultrahigh quality (UHQ), processed in an Elgastat UHQ II apparatus (Elga Ltd., High Wycombe, Bucks, England). Phosphate buffered saline (PBS) buffer was prepared from tablets from Sigma Aldrich (Ref. P4417) according to their instructions resulting in 137 mM NaCl, 2.7 mM KCl and 10 mM phosphate buffer solution (pH 7.4 at 25 C). Unless otherwise specified, any other chemicals used were at least of analytical grade.

All surfactants were used at a concentration well above the CMC (Critical Micelle Concentration) in PBS. More information on the CMC of each surfactant is found in Supplementary Information Section S1. The concentrations used were 2.5 time the CMC in water: 21.25 mM for SDS, 0.16 mM for C_12_E_5_ and 7.3 mM for CAPB.

### Saliva collection

Stimulated human whole saliva (HWS) was used for all experiments. The saliva was collected from two subjectively healthy adult donors, from whom informed consent was obtained, by chewing on parafilm^40^ while drooling into a chilled tube. After collection, saliva from the two donors was pooled and immediately used. Ethical approval was obtained from the committee of research ethics at Lund University (2018/42). All methods carried out in this work were performed in accordance with the Declaration of Helsinki.

### Silica surfaces

Silica was used as a substrate for salivary pellicles in all experiments. For force spectroscopy and ellipsometry experiments, p-Doped (boron) silicon wafers with a resistivity of 10–20 Ω∙cm (Semiconductor Wafer Inc., Taiwan) oxidized in an oxygen atmosphere were used. For Neutron Reflectivity (NR) measurements, single crystal silicon (100) blocks (8 × 5 × 2 cm, Siltronix, France) were used. AFM, ellipsometry and NR surfaces were cleaned by means of a standard RCA protocol, with 5:1:1 H_2_O:NH_3_:H_2_O_2_ at 80 °C for 10 min^41^, followed by 10 min plasma (for surfaces used in AFM and ellipsometry experiments) or ozone (for surfaces used in NR experiments) treatment. Quartz Crystal Microbalance with Dissipation (QCM-D) silica sensors (Q-Sense, Sweden) were cleaned instead with a gentler protocol consisting of (1) 5 min plasma treatment, (2) immersion into a Hellmanex II solution (2% v/v) for 10 min, (3) extensive rinsing with UHQ water and (4) 10 min plasma treatment. All cleaning procedures yielded hydrophilic surfaces with < 5° water contact angles.

### Quartz crystal microbalance with dissipation

Quartz Crystal Microbalance with Dissipation (QCM-D) measurements were performed using an E4 system (Q-sense AB, Sweden). A detailed description of the technique and its basic principles can be found elsewhere^42^. Briefly, an alternating-current voltage is applied through a gold-coated quartz chip to stimulate the shear mode oscillation of the quartz crystal. In our experiments, we used QCM-D surfaces where the gold was further coated with a silica layer (ref QSX 303, Q-sense AB, Sweden) that were further cleaned according to the protocol provided above.

Experiments consisted in the continuous monitoring of frequency and dissipation shifts for the different overtones of the sensors over the following experimental steps: (1) First, PBS buffer was flowed into the sensor chamber and allowed to stabilized until a stable baseline was achieved for both monitored signals. (2) Then, fresh HWS was flowed into the chambers for ~ 5 min and allowed to adsorb for additional ~ 55 min under non flow conditions. (3) This was followed by ~ 5 min of rinsing with PBS buffer followed by ~ 55 min of stabilization in the same conditions. (4) Next, surfactant solutions were flowed through the chamber for ~ 5 min before the flow is again stopped allowing the surfactants to interact with salivary pellicles a further ~ 55 min. (5) This was followed by a PBS rinsing step. Solutions were supplied into the QCM-D chamber using an Ismatec peristaltic pump IPC-N 4 at a flow rate of 0.1 mL·min^−1^.

The Q-Tools software (Q-Sense AB, Sweden) was employed for fitting data to the Voigt model (details on the fits are provided in Supplementary Information Section S2). Fitting to the Voigt model provides the layer thickness. In order to provide an areal mass value, we assumed a value for the pellicle density of 1.08 g/cm^3^^43^.

### Ellipsometry

Ellipsometry investigations were performed with a Rudolph thin film ellipsometer (type 43603-200E, Rudolph Research, USA) equipped with a Xenon light source filtered to 442.9 nm, with a setup based on null ellipsometry according to the principles of Cuypers^44^ and automated according to the concept of Landgren and Jönsson ^45^. Theoretical principles can be found elsewhere^46^. Samples were placed in a trapezoid cuvette made of optical glass (Hellma, Germany) equipped with a magnetic stirrer and temperature set to 25 °C. The determination of the silicon complex refractive index, and of the thickness and refractive index of the silicon oxide layer was performed using air and water as ambient media^45^. Four zone measurements were conducted to minimize systematic errors. Ellipsometry experiments were performed by means of similar steps to those followed in QCM-D investigations. However, in this case a flow velocity of 15 ml·min^−1^ was used. The adsorbed amount, Γ, was then calculated using de Feijter’s equation^47^:

1 $${\Gamma }\,\,=\,\,\frac{({n}_{f}{-n}_{o}){d}_{f}}{dn/dc}$$

where *n*_f_ is the refractive index of the adsorbed film, *d*_*f*_ its thickness, *n*_o_ the refractive index of the bulk solution and d*n*/d*c* is the refractive index increment as a function of bulk concentration for which a value of 0.18 ml/g was assumed ^47^.

### Force spectroscopy

A commercial Atomic Force Microscope (AFM) setup equipped with a liquid cell (MultiMode 8 SPM with a NanoScope V control unit, Bruker AXS, Santa Barbara CA) was utilized for the acquisition of force ramps. Rectangular silicon nitride levers with a nominal normal spring constant of 0.1 N·m^−1^ were employed in all the experiments (OMLC-RC800PSA, Olympus, Japan). Before every experiment, tips were rubbed against a clean freshly cleaved mica surface in PBS buffer, a procedure that leads to the removal of asperities and hence achievement of a smooth tip surface^48^.

Force ramps were obtained at different lateral positions by operating the AFM in the force volume (FV) mode ^49^ and analyzed with the FSAS software (https://git.io/JmEOS). Specifically, FV measurements consisted on 64 × 64 force ramps obtained at a speed of 1 µm·s^−1^ over an area of 2 µm × 2 µm. Analysis of force ramps was done following a process detailed in a previous work (^12^, Supplementary Information Section S3). Briefly, raw force ramps were first transformed into a deflection vs sample position representation by scaling a position sensitive photodetector signal by the slope of the contact region of force ramps obtained on a clean mica surface. Then, the contact point was obtained for each ramp by fitting its contact region with the Hertz contact model for a sphere-plane geometry and used as an offset for sample vertical position. Then, deflection was transformed into tip-sample interaction force by scaling the cantilever deflection by its spring constant (which was calculated for each cantilever by means of the Sader method ^50^). Force ramps were then converted into a force vs tip-sample distance representation. Finally, for analysis of steric interactions the non-contact region of the ramps (forces for a tip-sample distance d_ts_ > 5 nm) were fitted to an exponential function:

2 $${F}_{exp}={F}_{0}{e}^{\frac{{-d}_{ts}}{{\lambda }_{exp}}}$$

from where an exponential amplitude, F_0_, and a characteristic length, λ_exp_, indicative of the thickness of the steric repulsive layer ^30,31^ could be calculated.

For force spectroscopy experiments, human whole saliva (100 µl) was dropped onto clean hydrophilic silica surfaces and left to adsorb for 1 h. Then, surfaces were extensively rinsed with PBS buffer and placed immediately in the AFM. Subsequent treatment of the salivary pellicles with surfactants and corresponding rinsing steps with PBS buffer were performed in-situ following the same order as for QCM-D investigations.

### Neutron reflectivity

Neutron reflectivity (NR) measurements were performed on INTER (https://doi.org/10.5286/ISIS.E.RB1820559) and SURF (https://doi.org/10.5286/ISIS.E.RB1720420), horizontal time-of flight reflectometers at the ISIS neutron source (UK) and at SuperAdam (https://doi.org/10.5291/ILL-DATA.CRG-2539), a monochromatic machine (λ = 5.21 Å) with a horizontal scattering plane at Institut Laue-Langevin (France) ^51^. A q-range of 0.01 Å^−1^ to 0.3 Å^−1^, where $Q=\frac{4\pi }{\lambda }sin(\theta )$ was achieved using two angles of incidence; 0.7 and 2.3 on INTER. To achieve a similar Q-range, incidence angles of were 0.35, 0.65 and 1.5 were used on SURF whereas on SuperAdam this was achieved by measuring the reflected beam over a range of sample angles. The measured reflected intensity, I(Q), was normalised by the direct beam, I_0_, to achieve the absolute reflectivity, R(Q). PEEK solid liquid flow cells were used. The cells were cleaned by bath sonication in ethanol, in 1:1 ethanol/UHQ mixture and finally in UHQ. All experiments were performed at room temperature. Solutions were exchanged using an HPLC pump at flow rate of 2 mL/min. In all experiments, the footprint was controlled to only illuminate the region within the area of the surface covered in liquid.

The silicon blocks were initially characterized in PBS buffer prepared with both H_2_O (hPBS, SLD = − 0.56 × 10^–6^ Å^−2^), silicon matched water (0.38:0.62 D_2_O:H_2_O, smwPBS, SLD = 2.07 × 10^–6^ Å^−2^) and D_2_O (dPBS, SLD = 6.36 × 10^–6^ Å^−2^). Human Whole Saliva (HWS) was then injected into the cell, allowing it to adsorb for 60 min. After adsorption, the pellicles were rinsed with dPBS, and then characterized in dPBS, smwPBS and hPBS. Salivary pellicles were then exposed for one hour to surfactant solutions: CAPB (SLD = 0.36 × 10^–6^ Å^−2^), chain deuterated C_12_E_5_ (dC_12_E_5_, SLD = 3.76 × 10^–6^ Å^−2^) and fully hydrogenated C_12_E_5_ (hC_12_E_5_, SLD = 0.129·10^–6^ Å^−2^). In all cases, the surfactants were dissolved in PBS at a concentration of 2.5 times the value of the Critical Micelle Concentration (CMC) in water (Supplementary Information Section S1). The surfactant-treated pellicles were then rinsed with dPBS and characterized in dPBS, smwPBS and hPBS (except for CAPB, for which only dPBS and hPBS characterizations were performed). All measurements were carried out at room temperature.

## Supplementary Information

Supplementary Information.
