# Supplementary material for: Effect of nonionic and amphoteric surfactants on salivary pellicles reconstituted in vitro
Source: Sci Rep. 2021 Jun 21;11:12913. doi: 10.1038/s41598-021-92505-4 (PMC8217253; doi:10.1038/s41598-021-92505-4)
Supplement: Supplementary file 1 — Supplementary Information. [file 41598_2021_92505_MOESM1_ESM.pdf]

Effect of nonionic and amphoteric surfactants on salivary pellicles reconstituted in vitro

- Supplementary Information -

Hannah Boyd[1,†], Juan F. Gonzalez-Martinez[1,†], Rebecca J. L. Welbourn[2], Kun Ma[2], Peixun Li[2], Philipp Gutfreund[3], Alexey Klechikov[3,4], Thomas Arnebrant[1], Robert Barker[5] and Javier Sotres[1,*]

[1] Biomedical Science Department & Biofilms-Research Center for Biointerfaces, Malmö University, 20506 Malmö, Sweden

[2] ISIS Neutron & Muon Source, Rutherford Appleton Laboratory, Didcot OX11 0QX, U.K.

[3] Institut Laue Langevin, 71 avenue des Martyrs, Grenoble 38000, France.

[4] Department of Physics and Astronomy, Uppsala University, 75120 Uppsala, Sweden

[5] School of Physical Sciences, University of Kent, CT2 7NZ Canterbury, U.K.

[†] Both authors contributed equally to this work

[*] Corresponding author. E-mail: javier.sotres@mau.se

S1. Critical micellar concentration (CMC) of surfactants

The CMC in water (at 25°C) for each of the surfactants used in this work has been reported in the literature: 8.5 mM for SDS [1], 0.065 mM for $C_{12}E_5$ [2] and 2.92 mM for CAPB [3]. In our work, we exposed salivary pellicles to solutions in PBS buffer of these surfactants, in all cases with a surfactant concentration 2.5 time higher than the CMC in water. The CMC of the surfactants is known to lower with increasing ionic strength. Therefore, choosing these values ensured that the surfactant concentration was well above the CMC in PBS buffer i.e., the solvent used in this study.

Indeed, the CMC of SDS in PBS buffer was shown in literature to be 2mM [4]. However, we did not find this information for the CMC in PBS buffer of $C_{12}E_5$ or CAPB. In order to make sure that we worked at concentrations above the CMC in PBS buffer, we used the drop volume method for determining this quantity for both $C_{12}E_5$ and CAPB.

The method follows Tate's law;

$$W = 2\pi r\gamma \qquad \text{(Eq. S1)}$$

where W is the drop weight, r is the capillary radius and γ is the surface tension. When the weight, W, is equal to the right hand side of the equation, a drop falls from a small capillary. This can then be exploited to determine the surface tension of solutions. Above the CMC the surfactant will no longer cause the surface tension to lower. Therefore, by determining the concentration at which the surface tension is no longer affected, the CMC can be found.

The drop volume method is carried out on the Lauda drop volume tensiometer TVT, which consists of a 1ml syringe, with a plunger moved by a stepper motor so that the expelled volume (and mass) of the droplet can be accurately known. These droplets detach when the force of gravity exceeds that of the surface tension and knowing the drop volume (weight), the radius of the tip and the density of the liquid the surface tension can be calculated.

The surface tension for each concentration of surfactant was measured three times and averaged. The results are shown in Figure S1. The CMC in PBS buffer of $C_{12}E_5$ was found to be 0.022 mM and that of CAPB was 1.1 mM. These are both below the CMC in water.

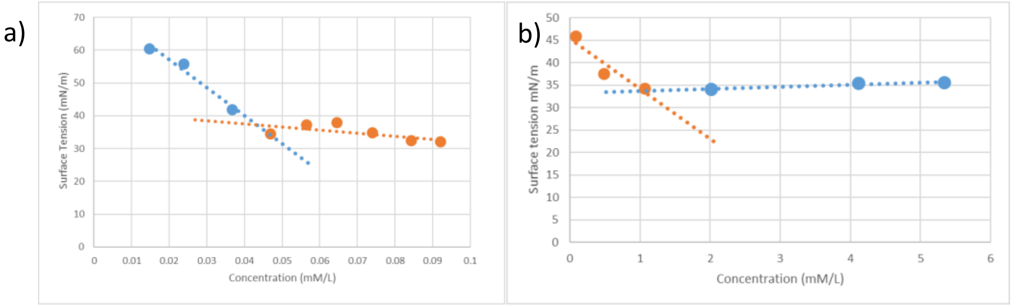

Figure S1. Surface tension measurements for solutions of **a)** $C_{12}E_5$ and **b)** CAPB in PBS buffer

S2. Voigt modelling of QCM-D data

The experimental outputs of QCM-D experiments are the shifts in the frequency of the resonance, f_n, and the dissipation factor, D_n, of each of the overtones of the sensor surface. If the adsorbed layer is rigid enough, the adsorbed amount and the shift in f_n are linearly related [5]. However, protein films may not be considered rigid, but viscoelastic instead. For viscoelastic films, where noticeable shifts in the dissipation factor, ΔD_n, are observed, more trustable data are obtained by applying the Voigt model. The expressions obtained by applying the Voigt model for a viscoelastic film adsorbed onto a solid substrate and immersed into a Newtonian fluid are [6]:

$$\Delta f_n / f_n = -\frac{d_p \rho_p}{d_Q \rho_Q}\left[1 - \eta_l \rho_l \frac{\left(\eta_p / \rho_p\right)\omega_n}{\mu_p^2 + \omega_n^2 \eta_p^2}\right] = -\frac{\Gamma_p}{d_Q \rho_Q}\left[1 - \eta_l \rho_l \frac{\left(\eta_p / \rho_p\right)\omega_n}{\mu_p^2 + \omega_n^2 \eta_p^2}\right] \quad \text{(Eq. S2a)}$$

$$\Delta D_n = \frac{1}{d_Q \rho_Q}\left[\eta_l \rho_l \frac{d_p\, \mu_p \omega_n^2}{\mu_p^2 + \omega_n^2 \eta_p^2}\right] \quad \text{(Eq. S2b)}$$

In these equations d, ρ, and ω_n stand for thickness, density, and $2\pi f_n$ respectively. The subscripts Q, p, and l stand for quartz crystal, protein film, and liquid medium respectively. By a numerical fit of the frequency and the dissipation values obtained at three different overtones (n = 5, 7 and 9) not only the adsorbed amount, Γ, can be obtained, but also the viscoelastic properties of the film, like the shear elastic modulus, μ, and the shear viscosity η [7].

We have used the Voigt model to fit our experimental data and for this purpose we employed the Q-Tools software (Q-Sense AB, Sweden). Initial input values given for fluid density and viscosity, and for the density of the adsorbed protein films are specified below.

**Fixed Parameters**		**Parameters to fit**	
Fluid density (kg/m^3)	1000	Layer η (Pa·s)	0.0001 – 0.01
Fluid η (Pa·s)	0.001	Layer μ (Pa)	$10^3 – 10^7$
Layer density (kg/m^3)	1080	Layer d (m)	$10^{-10} – 10^{-6}$

S3. Analysis of force spectroscopy data

Raw force ramps consisted in a set of photodetector signals for different sample vertical positions values (Fig. S3a). The offset for the photodetector signal was first found out as the average of values far from the contact. Then, this signal was converted into cantilever deflection values by multiplying by a factor obtained from a linear fit of the contact region of force ramps obtained on clean mica surfaces.

The contact point i.e., offset for the sample vertical values was found by fitting the contact region of the force ramp to the Hertz model as detailed in [8] (Fig. S3b). Finally, the cantilever deflection signal was converted into tip-sample interaction force by multiplying by the cantilever elastic force constant determined by means of the Sader method [9], and the sample vertical positions transformed into tip-sample distance values (d_{ts}) by adding to the former the cantilever deflection (Fig. S3c). Finally, the noncontact region of this representation (d_{ts}>5nm) was fitted to an exponential function (Fig. S3c).

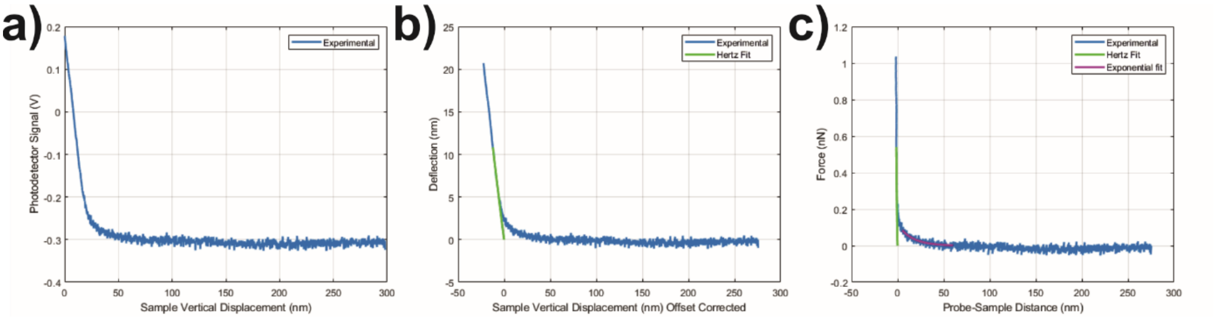

Figure S3. a) Raw force ramp obtained on a salivary pellicle in PBS buffer. **b)** Same force ramp in a deflection vs sample vertical displacement representation where the contact point by fitting the contact region to the Hertz model. **c)** Same force ramp in a force vs probe-sample distance representation where the non-contact region was fitted to an exponential function.

S4. QCM-D experiments for the salivary pellicle treated with SDS

QCM-D was used to cofirm previous ellipsometry studies which have shown SDS to cause a complete desorption of the pellicle. Figure S4 shows the adsorption of Human Whole Saliva (HWS) causes a frequency shift from the baseline (0Hz) to -65Hz (7th overtone) in the bulk and -60Hz after rinsing with PBS. This clearly shows an adsorbed protein layer, however, after SDS is introduced into the cell, the frequency immediately returns to zero indicating the entire film has been removed.

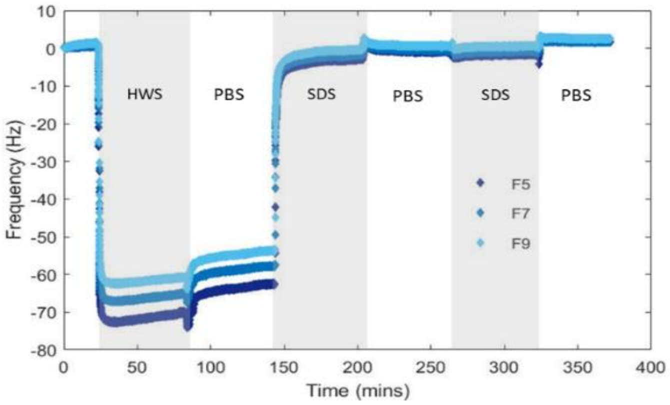

Figure S4. Frequency shifts monitored using QCM-D (5th, 7th and 9th overtones) for the acquired pellicle before and after SDS/PBS rinsing cycles as described in the main text.

S5. Thickness and refractive index values from ellipsometry

Figure S5 shows thickness and refractive indices for the experiment represented in Fig. 2 of the manuscript. Overall, these data support that exposure of salivary pellicles to CAPB resulted in lower thickness and higher density than exposure to $C_{12}E_5$.

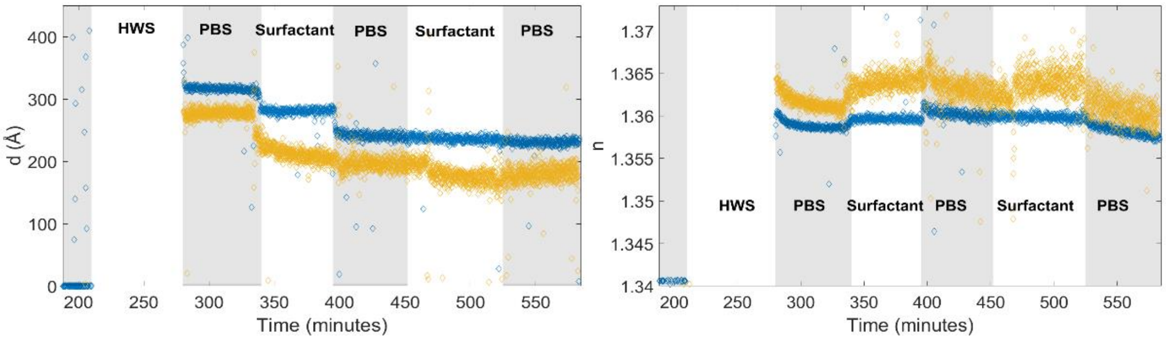

Figure S5. a) Thickness and **b)** refractive index values for the same experiments as those represented in Fig. 2 of the main manuscript where salivary pellicles on hydrophilic silica surfaces were exposed to $C_{12}E_5$ (blue) and CAPB (yellow) solutions.

S6. Atomic Force Microscopy of salivary pellicles before and after exposure to surfactants

Salivary Pellicles before and after treatment with $C_{12}E_5$ and CAPB surfactants were visualized by means of an Atomic Force Microscope (AFM) operated in the Peak Force Tapping Mode (Fig. S6). It can be observed that salivary pellicles and salivary pellicles treated with $C_{12}E_5$ exhibited planar homogeneous surfaces. However, pellicles treated with CAPB exhibited features with lateral sizes in the order of ~µm and heights in the order of ~20nm occupying ~5% of the surface area.

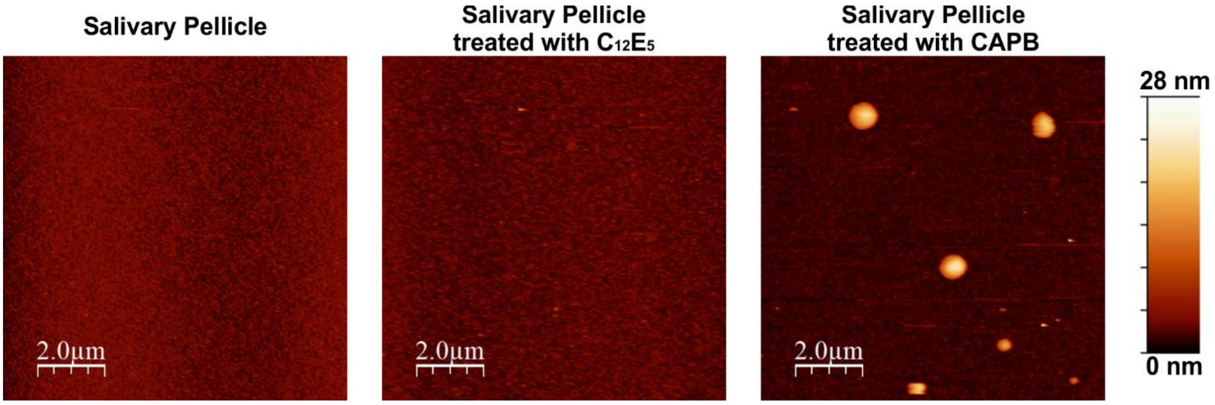

Figure S6. AFM images in PBS buffer of representative salivary pellicles before and after treatment with $C_{12}E_5$ and CAPB.

S7. Detailed neutron reflectivity analysis

We used the refnx software [10] for the analysis of NR data. In all cases, the interface between the silicon support and the bulk solvent was considered as a stratified medium composed by different slabs. For fitting the clean silicon blocks, we used a Si/SiO2/Solvent structure. For salivary pellicles, we considered a two-layer structure successfully used in previous works [8, 11]. Thus, for fitting their NR profiles we used a Si/SiO2/InnerLayer/OuterLayer/Solvent structure.

For fitting the pellicles treated with surfactants ($dC_{12}E_5$, $hC_{12}E_5$ and CAPB) we used a similar model as for non-treated salivary pellicles. However, in these cases we considered that the treated pellicles might include a percentage of surfactant:

$$SLD_{treated-layer} = (SLD_{nontreated-layer}\ x\ Coverage_{salivary\ components}) + (SLD_{surfactant}\ x\ Coverage_{surfactant})$$

Fits for the experimental data and parameters found from these fits follow in the pages below.

Effect of $dC_{12}E_5$ on salivary pellicles

Reflectivity data in different contrasts and corresponding fits for a clean silicon block, a salivary pellicle adsorbed on the same block and the same salivary pellicle after being treated with a $dC_{12}E_5$ solution are shown below.

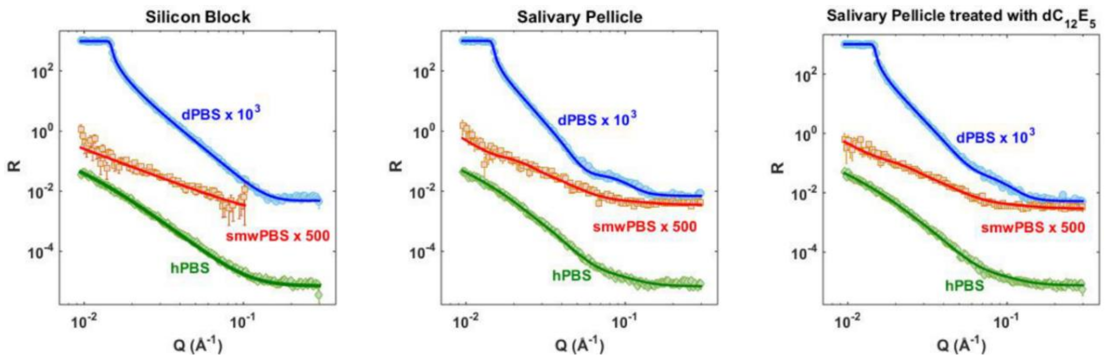

Fit Parameters for the non-treated salivary pellicle:

Layer	SLD (10^{-6} Å^{-2})		Thickness (Å)	Roughness (Å)	Hydration (%)
**Si**	2.07		-	-	-
**SiO_2**	3.47		11±1	2±1	22.4±1.2
**Pellicle Inner Layer**	dPBS	2.53±0.05	46±1	4±1	62.0±0.8
	smwPBS	2.51±0.07			
	hPBS	1.26±0.06			
**Pellicle Outer Layer**	dPBS	5.12±0.18	286±12	15±1	97.1±0.2
	smwPBS	3.10±0.60			
	hPBS	2.20±1.30			
**Solvent**	dPBS	6.36	-	52±13	-
	smwPBS	2.07			
	hPBS	-0.56			

Fit Parameters for the salivary pellicle treated with a 2.5 CMC $dC_{12}E_5$ in PBS solution:

Layer		Proportion (%)	SLD (10^{-6} Å^{-2})		Thickness (Å)	Roughness (Å)	Hydration (%)
**Si**		-	2.07		-	-	-
**SiO_2**		-	3.47		11±1	2±1	25.0±9.0
**Pellicle Inner Layer**	**Salivary Content**	92±2	dPBS	2.53±0.05	45±1	4±1	70.1±0.5
			smwPBS	2.51±0.07			
			hPBS	1.26±0.06			
	**$dC_{12}E_5$**	8±2	3.76				
**Pellicle Outer Layer**	**Salivary Content**	96±5	dPBS	5.12±0.18	277±16	15±1	98.0±0.1
			smwPBS	3.10±0.60			
			hPBS	2.20±1.30			
	**$dC_{12}E_5$**	4±5	3.76				
**Solvent**		-	dPBS	6.36	-	54±15	-
			smwPBS	2.07			
			hPBS	-0.56			

Effect of $hC_{12}E_5$ on salivary pellicles

Reflectivity data in different contrasts and corresponding fits for a clean silicon block, a salivary pellicle adsorbed on the same block and the same salivary pellicle after being treated with a $hC_{12}E_5$ solution are shown below.

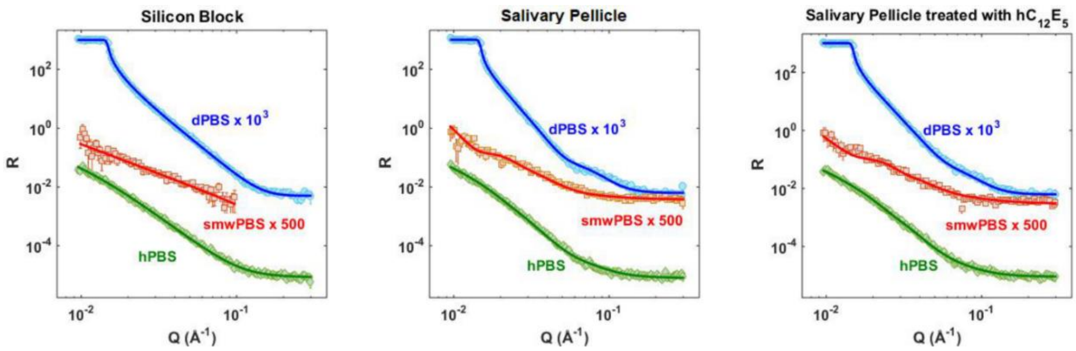

Fit Parameters for the non-treated salivary pellicle:

Layer	SLD (10^{-6} Å^{-2})		Thickness (Å)	Roughness (Å)	Hydration (%)
**Si**	2.07		-	-	-
**SiO_2**	3.47		10±1	2±1	25±9
**Pellicle Inner Layer**	dPBS	2.90±0.40	48±1	2±1	57.6±0.3
	smwPBS	2.70±0.04			
	hPBS	1.59±0.13			
**Pellicle Outer Layer**	dPBS	5.70±0.50	297±10	23±1	92.7±0.5
	smwPBS	3.60±0.20			
	hPBS	2.80±1.60			
**Solvent**	dPBS	6.36	-	85±9	-
	smwPBS	2.07			
	hPBS	-0.56			

Fit Parameters for the salivary pellicle treated with a 2.5 CMC $hC_{12}E_5$ in PBS solution:

Layer		Proportion (%)	SLD (10^{-6} Å^{-2})		Thickness (Å)	Roughness (Å)	Hydration (%)
**Si**		-	2.07		-	-	-
**SiO_2**		-	3.47		10±1	2±1	25.0±9.0
**Pellicle Inner Layer**	**Salivary Content**	93±4	dPBS	2.90±0.40	42±1	2±1	63.2±1.2
			smwPBS	2.70±0.04			
			hPBS	1.59±0.13			
	**$hC_{12}E_5$**	7±4	0.13				
**Pellicle Outer Layer**	**Salivary Content**	94±5	dPBS	5.70±0.50	280±9	23±1	96.7±0.3
			smwPBS	3.60±0.20			
			hPBS	2.80±1.60			
	**$hC_{12}E_5$**	6±5	0.13				
**Solvent**		-	dPBS	6.36	-	46±12	-
			smwPBS	2.07			
			hPBS	-0.56			

Effect of CAPB on salivary pellicles

Reflectivity data in different contrasts and corresponding fits for a clean silicon block, a salivary pellicle adsorbed on the same block and the same salivary pellicle after being treated with a CAPB solution are shown below.

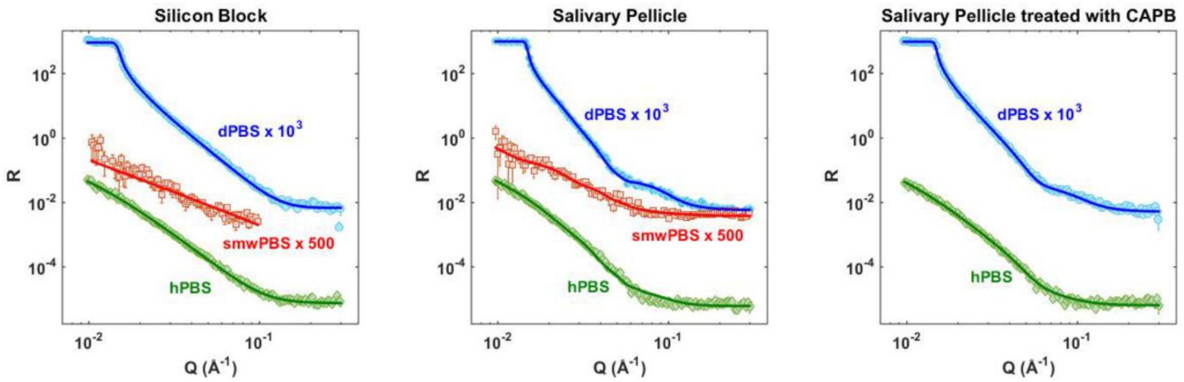

Fit Parameters for the non-treated salivary pellicle:

Layer	SLD (10^{-6} Å^{-2})		Thickness (Å)	Roughness (Å)	Hydration (%)
**Si**	2.07		-	-	-
**SiO_2**	3.47		11±1	4±1	45.0±3.0
**Pellicle Inner Layer**	dPBS	2.55±0.03	46±1	2±1	55.7±0.3
	smwPBS	2.48±0.02			
	hPBS	1.59±0.03			
**Pellicle Outer Layer**	dPBS	4.84±0.13	325±11	21±1	97.5±0.2
	smwPBS	2.70±0.40			
	hPBS	1.70±0.70			
**Solvent**	dPBS	6.36	-	19±14	-
	smwPBS	2.07			
	hPBS	-0.56			

Fit Parameters for the salivary pellicle treated with a 2.5 CMC CAPB in PBS solution:

Layer		Proportion (%)	SLD (10^{-6} Å^{-2})		Thickness (Å)	Roughness (Å)	Hydration (%)
**Si**		-	2.07		-	-	-
**SiO_2**		-	3.47		11±1	4±1	45.0±3.0
**Pellicle Inner Layer**	**Salivary Content**	99.4±0.7	dPBS	2.55±0.03	35±1	2±1	54.4±0.9
			hPBS	1.59±0.03			
	**CAPB**	0.6±0.7	0.38				
**Pellicle Outer Layer**	**Salivary Content**	92.0±2.0	dPBS	4.84±0.13	117±8	20±1	95.5±0.5
			hPBS	1.70±0.70			
	**CAPB**	8.0±2.0	0.38				
**Solvent**		-	dPBS	6.36	-	24±3	-
			hPBS	-0.56			

Supplementary Information References

1. Domínguez, A., et al., *Determination of critical micelle concentration of some surfactants by three techniques.* J. Chem. Educ., 1997. **74**(10): p. 1227.
2. Cuny, V., et al., *Structural properties and dynamics of C12E5 molecules adsorbed at water/air interfaces: A molecular dynamic study.* Colloids Surf., A Physicochem. Eng. Asp., 2008. **323**(1-3): p. 180-191.
3. Dai, C., et al., *Adsorption behavior of cocamidopropyl betaine under conditions of high temperature and high salinity.* J. Appl. Polym. Sci., 2014. **131**(12).
4. Berg, I.C.H., et al., *Salivary protein adsorption onto hydroxyapatite and sds-mediated elution studied by in situ ellipsometry.* Biofouling, 2001. **17**(3): p. 173-187.
5. Sauerbrey, G., *Verwendung von schwingquarzen zur wägung dünner schichten und zur mikrowägung.* Zeitschrift für Physik 1959. **155**: p. 206-222.
6. Macakova, L., E. Blomberg, and P.M. Claesson, *Effect of Adsorbed Layer Surface Roughness on the QCM-D Response: Focus on Trapped Water.* Langmuir, 2007. **23**(24): p. 12436-12444.
7. Voinova, M.V., et al., *Viscoelastic Acoustic Response of Layered Polymer Films at Fluid-Solid Interfaces: Continuum Mechanics Approach.* Physica Scripta, 1999. **59**(5): p. 391-396.
8. Boyd, H., et al., *A comparison between the structures of salivary pellicles and oral mucin (MUC5B) films.* J. Colloid Interface Sci., 2021. **584**: p. 660-668.
9. Sader, J.E., J.W.M. Chon, and P. Mulvaney, *Calibration of rectangular atomic force microscope cantilevers.* Rev. Sci. Instrum., 1999. **70**(10): p. 3967-3969.
10. Nelson, A.R.J. and S.W. Prescott, *refnx: neutron and X-ray reflectometry analysis in Python.* J. Appl. Cryst., 2019. **52**: p. 193-200.
11. Cárdenas, M., et al., *Human saliva forms a complex film structure on alumina surfaces.* Biomacromolecules, 2007. **8**(1): p. 65-69.
